# Comparison of Combined and Sequential Surgery for Proliferative Diabetic Retinopathy: A Single Surgeon Study

**DOI:** 10.1371/journal.pone.0108933

**Published:** 2014-09-30

**Authors:** Ye Yang, Jie Zhang, Hong Yan

**Affiliations:** 1 Department of Ophthalmology, Tangdu Hospital, Fourth Military Medical University, Xi'an, China; 2 Chinese People's Liberation Army 451 Hospital, Xi'an, Shaanxi, China; Massachusetts Eye & Ear Infirmary, Harvard Medical School, United States of America

## Abstract

**Purpose:**

To compare the results of combined and consecutive surgeries to treat proliferative diabetic retinopathy and cataract.

**Methods:**

Retrospective comparative study. Forty-one patients with proliferative diabetic retinopathy (PDR) were enrolled. Twenty-nine eyes for the combined surgery group and twelve eyes for the sequential group were included. All surgeries were performed by one surgeon. Phacoemulsification was performed using a clear cornea incision. The vitrectomy was performed using a 20-gauge vitreous cutter.

**Results:**

The best corrected visual acuity (BCVA) and intra- and post-operative complications were the main outcome measures. In the combined surgery group, the BCVA increased in 18 (62.1%) eyes, while eight (27.6%) eyes remained stable and three (10.3%) eyes decreased. Postoperative complications included fibrinous exudation in nine eyes, macular edema in three eyes and vitreous hemorrhage in three eyes. In the sequential surgery group, the BCVA increased in seven (58.3%) eyes, remained the same in four (33.3%) eyes and was reduced in one (8.3%) eye. Postoperative complications included macular edema in two eyes, neovascular glaucoma in two eyes and vitreous hemorrhage in one eye.

**Conclusions:**

Both combined and sequential surgeries are safe and effective for treating PDR and cataracts. The combined surgery had a higher incidence of fibrinous exudation.

## Introduction

Vitrectomy has been widely used to treat proliferative diabetic retinopathy (PDR). The most common complication of pars plana vitrectomy (PPV) is the nuclear sclerosis cataract, which is reported in 75%–95% of cases within two years of surgery [1], [2], [3]. Age, pre-existing nuclear sclerosis and intra-operative use of silicone oil and gas are the risk factors for cataract formation [4]. Many diabetic patients also have different degrees of cataract before vitrectomy.

Whether to remove the crystalline lens during vitreous surgery of diabetic patients is controversial. Cataract surgery after vitrectomy is technically demanding, due to the loss of vitreous support and posterior capsule weakness [5]. Moreover, removal of the lens ensures better visualization of anterior vitreous structures and the retina. Therefore, combined vitrectomy and phacoemulsification surgery eliminates the inconvenience of a second surgery and shortens the mean recovery time [6]. Nevertheless, an increased incidence of postoperative anterior segment neovascularization after the combined surgery was reported in some studies [7], [8]. While many surgeons believed that the crystalline lens provided a barrier that had protective effects on the anterior segment and retina, with the improvement of surgical instrumentation and use of intraoperative laser photocoagulation, surgeons have started to remove the lens in combination with PPV, and there are several reports of the safety of this combined technique in the treatment of PDR [4], [6], [9], [10].

However, no study has made a direct comparison between the combined and sequential surgery for diabetic retinopathy performed by one surgeon. The purpose of this research was to compare the complications and visual acuity outcomes of the combined and sequential surgery.

## Methods

The study was approved by the Research Ethics Committee of the Fourth Military Medicine University (Xi'an, China). The data were analyzed anonymously. All participants were informed about possible complications and provided written informed consent prior to surgery.

The medical records of 29 patients who underwent vitrectomy combined with phacoemulsification and 12 patients with phacoemulsification subsequent to vitrectomy were retrospectively reviewed. Combined surgeries were carried out between January 2008 and March 2011. In the sequential surgery group, vitrectomy was performed between January 2007 and December 2010. All surgeries were performed by the same surgeon (Dr. Y.H.), who is experienced in both vitreous and phacoemulsification surgeries.

The median age of patients was 63 years (ranging from 52 to 71 years) in the combined surgery group, and 55 years (ranging from 45 to 67 years) in the sequential surgery group. In the two groups, slightly more than half the patients were male [combined: 16 (55.2%), sequential: 7 (58.3%)]. The followed up time of all patients was more than six months. In the sequential group, the median interval between the two surgeries (vitrectomy and phacoemulsification) was nine months.

Study inclusion criteria were vitreous hemorrhage (VH) and fiber proliferation. Patients with extensive macular traction, rubeosis iridis, neovascular glaucoma and advanced tractional retinal detachment were excluded. All patients underwent combined or sequential surgeries under local anesthesia. The indication for combined surgery was judged by the surgeon, based on the degree of cataract and difficulty of obtaining good visualization during the surgery. Phacoemulsification was performed using a clear cornea incision (3.2 mm), and a foldable acrylic intraocular lens was implanted in the capsular bag. A standard three-port pars plana vitrectomy was performed using a 20-gauge vitreous cutter (Megatron, Germany). In the combined group, phacoemulsification was carried out before the vitreoretinal procedure. The mean surgical time was 88 minutes. In the sequential group, the mean operation time was 55 minutes for vitrectomy and 18 minutes for phacoemulsification. Mean follow-up time was 13 months (ranging from 6 to 36 months).All patients provided written consent before surgery after discussing the benefits and risks of the procedure.

We recorded the following data: patient age, gender, diabetes duration, HbA1c, degree of vitreoretinal pathology, preoperative and postoperative visual acuity and intraoperative and postoperative complications. Intraoperative complications included dislocation of the IOL, posterior capsule tears, and anterior chamber hemorrhage. Postoperative complications comprised fibrinous exudation in the anterior chamber, formation of posterior synechia, silicone oil prolapse in the anterior chamber, and endophthalmitis.

Statistical analysis of postoperative complications and final visual acuity was performed using Fisher's exact probability test. *P* value<0.05 was considered to be statistically significant.

## Results

The demographic data are shown in Table 1. Most factors were similar between the two groups, including sex, diabetes duration, glycosylated hemoglobin, Indications and BCVA. We acknowledge that for the patients' benefit we selected cases with more severe cataracts for combined surgery, which resulted in an age difference between the two groups. The mean follow up time of the combined surgery and sequential surgery groups was 12.9 and 14.3 months, respectively. In the sequential surgery group, all eyes showed progression of cataract after surgery. Cataract extraction was performed on average nine months after the vitrectomy surgery. The preoperative best corrected visual acuity (BCVA) ranged from 0.1 to perception of light in both groups. The postoperative BCVA ranged from 0.5 to hand movement in the combined group, and 0.4 to perception of light in the sequential surgery group.

**Table 1:** Patient demographic data.

	Combined group N = 29	Consecutive group N = 12
Age(years)	63 (52–71)	55 (45–67)
Sex (male)	16 (55.2%)	7 (58.3%)
Diabetes duration(years)	6.9±4.3	5.8±3.6
HbA1c (%)	8.24±1.5	7.35±1.2
Indications		
Retinal detachment	18	7
Vitreous hemorrhage	11	5
BCVA		
pre-operation	0.1- PL	0.1-PL
post-operation	0.5-HM	0.4-PL

HbA1c: Glycosylated hemoglobin; BCVA: Best corrected visual acuity; PL: Perception of light; HM: Hand movement.

The statistical analyses of endotamponades, endolaser, cryocoagulation and TA injection in vitrectomy are shown in Table 2. The percentages of the various operations were similar in the two groups.

**Table 2:** Endotamponades and other procedures in vitrectomy.

	Combined group N = 29	Consecutive group N = 12	P
Endotamponades			
C3F8 gas	5	3	0.568
Silicone oil	8	4	0.713
BSS	16	5	0.431
Endolaser	7	4	0.545
Cryocoagulation	4	2	0.813
Endolaser and cryocoagulation	4	1	0.627
TA injection	7	1	0.245

TA: Triamcinolone acetonide.

In the combined group, 18 (62.1%) eyes had a BCVA improvement of two or more lines, eight (27.6%) eyes remained the same and three (10.3%) eyes decreased. In the sequential surgery group, seven (58.3%) eyes had vision improvement of two or more lines, four (33.3%) eyes remained unchanged, and one (8.3%) eyes decreased. There was no statistically significant difference between the two groups (Table 3).

**Table 3:** Best corrected visual acuity.

BCVA	Combined group N = 29	Consecutive group N = 12	P
Improved≥2 lines	18 (62.1%)	7 (58.3%)	0.823
Within 2 lines	8 (27.6%)	4 (33.3%)	0.712
Decreased≥2 lines	3 (10.3%)	1 (8.3%)	0.843

BCVA: Best corrected visual acuity.

The postoperative complications are described in Table 4. Complications occurred in 15 of 29 patients (51.7%) in the combined surgery group and 4 of 12 patients (33.3%) in the sequential surgery group (*P*>0.05). Fibrinous exudation was the most common complication, which was found in nine eyes in the combined surgery group and in no eyes in the sequential group. The incidence rate was significantly higher (*P*<0.05) in the combined surgery group. Macular edema was detected in three eyes in the combined surgery group and two eyes in the sequential surgery group. Postoperative recurrent vitreous hemorrhage was found in three eyes from the combined surgery group and in one eye from the sequential surgery group. Cystoid macular edema was found in one patient in the combined group, while an intra-operative posterior capsular tear was found in one eye of the sequential group. Other complications included neovascular glaucoma (NVG) in one eye in each of the combined and sequential groups, prepupillary membrane in one eye of the combined group, retina detachment in one eye of the combined group and posterior capsule opacification in one eye in each of the combined and sequential groups. There was no significant difference in these surgical complications between the two groups (*P*>0.05). No endophthalmitis occurred in either group.

**Table 4:** Post-operative complications.

Complications	Combined group N = 29	Consecutive group N = 12	P
Fibrinous exudation	9	0	0.029*
Macular Edema	3	2	0.574
Pupillary membrane	1	0	0.515
Neovascular glaucoma	1	1	0.509
Vitreous hemorrhage	3	1	0.843
Retina detachment	1	0	0.515
Posterior Capsule Opacification	1	1	0.509

* *P*<0.05.

## Discussion

In this study we found no significant difference between the two groups in visual acuity improvement after surgery. In both groups more than half of the patients exhibited a BCVA improvement over two lines. This result is similar to a previous study by Rivas *et al*. [4].

A statistically higher percentage was found in the combined surgery group of cases showing fibrinous exudation in the anterior chamber. And yet it was interesting that the macular edema rates did not. The different rates of fibrinous exudation in the two groups may be due to the longer operation time, lack of red reflex and the difficulty of circular capsulorhexis in the combined surgery. Moreover, to ensure the patients' benefit we selected cases with more severe cataracts for combined surgery. This may also increase the difficulty and operation time of the cataract surgery, leading to a higher rate of fibrinous exudation in the combined group. Finally, as shown in Table 2, a trend of more intra-vitreous TA injection during the operation may also contributed to reducing the macular edema rates in the combined group. However, previous authors reported different results in the ratio between the combined and sequential surgery groups [4], which may be due to the smaller number of cases included in both studies. As such, future studies involving larger numbers of cases are expected to determine more exactly the difference between the two surgical techniques.

Neovascular glaucoma occurred with a similar frequency in both groups of this study, and was medically controlled with topical anti-glaucomatous drugs. Only one patient required filtrating surgery in the consecutive surgery group. This complication was described in the literature as being associated with the combined procedure [11]. While previous studies noted that the incidence of anterior segment neovascularization was likely to increase after cataract operation in eyes with PDR [12], [13], [14], we observed no significant difference between the two groups in this study, which may be attributable to the development and improvement of modern vitreoretinal surgical techniques and intraocular endophotocoagulation [15], [16], [17]. In this study, endophotocoagulation was performed intraoperatively, which could reduce the incidence of postoperative neovascular glaucoma. Advances in systemic diabetes control may also facilitate the lower incidence of neovascular glaucoma [18]. Another theory suggests that an increase in vascular permeability factors, including vascular endothelial growth factor and interleukin-6, promote the development of neovascular glaucoma [19], [20]. Complete removal of the vitreous that contains internal growth factors may contribute to the decrease in the incidence of postoperative iris and angle neovascularization. Some researchers found a significantly higher incidence of postoperative recurrent VH and iris NV in the vitrectomy alone surgery group compared to the combined surgery group, which indicates that combined procedures in diabetic patients may be a better alternative to separate procedures or taking no action [21]. Differences in other post-operative complications such as macular edema, prepupillary membrane, opacification posterior capsule tear and retina detachment were not significant.

In our series, all phakic eyes showed some degree of nuclear sclerosis progression after surgery. The diabetic eye is more vulnerable to surgical trauma and thus consecutive surgeries can be difficult and technologically demanding. The loss of vitreous support can also result in increased occurrence of deepened anterior chamber, fragile zonule, flaccid posterior capsule, and increased rupture percentage [5].

Combined surgery shows many advantages for the therapy of diabetic retinopathy. Lens removal ensures better visualization of anterior vitreous structures and simplifies adequate peeling of the epiretinal membrane. In addition, if small retinal tears appear, they can be easily found and treated. Moreover, sufficient pan-retinal photocoagulation can be performed to decrease the formation of retina and iris neovascularization. Combined surgery also offers convenience for the patient and reduced costs, especially for patients from rural areas as they need only travel to the hospital for a single surgery. The combined surgery can diminish overall surgical costs as seen from a comparison of costs for the surgical procedures in both groups. In the sequential group, the cost for vitrectomy and phacoemulsification was 2,740 and 1,970 Chinese Yuan, respectively, and the total cost was about 15,300 Yuan. In the combined surgery group, the cost for the combined surgical procedures was 4,710 Yuan and the mean total cost was about 12,600 Yuan. However, it should be noted that this cost analysis does not consider indirect costs, including items such as follow-up visits and transportation.

In this study the mean recovery time of patients in the combined group was nine months shorter than that of the consecutive group, possibly because of cataract progression after vitreous surgery. This result was similar to a report by Muselier *et al.*, which showed a shorter recovery time for the combined surgery group in terms of visual acuity improvement after surgery for macular holes [22].

However, there are some disadvantages of the combined surgery such as increased surgical time, absence or reduction of the red reflex, increasing risk of surgical wound dehiscence, intra-operation myosis and bleeding of anterior structures. Moreover, cataract surgery may also worsen diabetic retinopathy in diabetic patients [23]. A longer duration of surgical intervention, a temporary elevation of intraocular pressure (IOP), a greater intensity of post-operative inflammation and a higher prevalence of cystoid macular edema have been reported in diabetic eyes [4], although in this study postoperative cystoid macular edema was found in only one patient in the combined group.

The present study is a single-surgeon study, in which the surgeries were performed by one surgeon using the same surgical approaches to lens management and vitrectomy surgery. We acknowledge that there may be limitations in our study. Here, indications for combined surgery were judged by the surgeon. For the patients' benefit, we selected cases with more severe cataract for combined surgery, which resulted in an age difference between the two groups. The retrospective and non-randomized nature of this study may thus weaken the conclusions. And because of the small sample size of this research, the results may have its limitations. Therefore, prospective randomized studies with larger numbers of patients should be performed in the future.

In this study we also want to look at all phakic patients who underwent surgery for PDR and then compare those who underwent initial ppv vs. those who underwent initial ppv/cataract extraction. However, because our hospital is a comprehensive tertiary hospital and PPV do not carry out in most primary hospitals, many patients who underwent PPV in our hospital were out-of-town. In the western region in China, the economic condition of many patients is poor. For the patients' convenience and economic reasons, some patients of other provinces who did not need cataract surgery after PPV were followed in local hospitals. And some patients gave up treatment for economic or other unknown reasons. So the complete data of these patients was difficult to collect. According to our observations, most patients followed in our hospital needed to undergo cataract surgery after PPV.

In conclusion, both combined surgery and consecutive surgery are safe and effective for the treatment of PDR and cataract. The visual outcomes of combined surgeries were similar to that of consecutive surgeries in this study. However, combined surgery has many advantages, such as faster visual recovery, which will reduce the patients' course of disease and reduce the total cost. As combined surgery offers significant convenience and cost savings, it can be performed on more patients with PDR and cataract.

## References

[1] Thompson JT, Glaser BM, Sjaarda RN, Murphy RP (1995) Progression of nuclear sclerosis and long-term visual results of vitrectomy with transforming growth factor beta-2 for macular holes. Am J Ophthalmol 119 (1) 48–54. 7825689 10.1016/s0002-9394(14)73812-7

[2] Smiddy WE, Feuer W (2004) Incidence of cataract extraction after diabetic vitrectomy. Retina 24 (4) 574–581. 15300079 10.1097/00006982-200408000-00011

[3] Chang MA, Parides MK, Chang S, Braunstein RE (2002) Outcome of phacoemulsification after pars plana vitrectomy. Ophthalmology 109 (5) 948–954. 11986103 10.1016/s0161-6420(01)01010-7

[4] Rivas-Aguiño P, García-Amaris RA, Berrocal MH, Sánchez JG, Rivas A, et al (2009) Pars plana vitrectomy, phacoemulsification, and intraocular lens implantation for the management of cataract and proliferative diabetic retinopathy: comparison of a combined versus two-step surgical approach. Arch Soc Esp Oftalmol 84 (1) 31–38. 19173136 10.4321/s0365-66912009000100005

[5] Treumer F, Bunse A, Rudolf M, Roider J (2006) Pars plana vitrectomy, phacoemulsification and intraocular lens implantation. Comparison of clinical complications in a combined versus two-step surgical approach. Graefes Arch Clin Exp Ophthalmol 244 (7) 808–815. 16328429 10.1007/s00417-005-0146-9

[6] Lahey JM, Francis RR, Kearney JJ (2003) Combining phacoemulsification with pars plana vitrectomy in patients with proliferative diabetic retinopathy: a series of 223 cases. Ophthalmology 110 (7) 1335–1339. 12867387 10.1016/S0161-6420(03)00454-8

[7] Blankenship GW (1980) The lens influence on diabetic vitrectomy results. Report of a prospective randomized study. Arch Ophthalmol 98 (12) 2196–2198. 7447772 10.1001/archopht.1980.01020041048008

[8] Schachat AP, Oyakawa RT, Michels RG, Rice TA (1983) Complications of vitreous surgery for diabetic retinopathy: Postoperative complications. Ophthalmology 90 (5) 522–529. 6192378 10.1016/s0161-6420(83)34540-1

[9] Vatavuk Z, Bencić G, Loncar VL, Petric I, Mandić Z (2005) Phacoemulsification, Vitrectomy and the Implantation of an Intraocular Lens in Diabetic Patients. Coll. Antropol 29 Suppl. 1: 13–16. 16193668

[10] Canan H, Sizmaz S, Altan-Yaycioğlu R (2013) Surgical results of combined pars plana vitrectomy and phacoemulsification for vitreous hemorrhage in PDR. Clin Ophthalmol 7: 1597–1601. 23966765 10.2147/OPTH.S47780 PMC3745294

[11] Demetriades AM, Gottsch JD, Thomsen R, Azab A, Stark WJ, et al (2003) Combined phacoemulsification, intraocular lens implantation, and vitrectomy for eyes with coexisting cataract and vitreoretinal pathology. Am J Ophthalmol 135 (3) 291–296. 12614744 10.1016/s0002-9394(02)01972-4

[12] Poliner LS, Christianson DJ, Escoffery RF, Kolker AE, Gordon ME (1985) Neovascular glaucoma after intracapsular and extracapsular cataract extraction in diabetic patients. Am J Ophthalmol 100 (5) 637–643. 2414993 10.1016/0002-9394(85)90617-8

[13] Pavese T, Insler MS (1987) Effects of extracapsular cataract extraction with posterior chamber lens implantation on the development of neovascular glaucoma in diabetics. J Cataract Refract Surg 13 (2) 197–201. 2437281 10.1016/s0886-3350(87)80135-9

[14] Sadiq SA, Chatterjee A, Vernon SA (1995) Progression of diabetic retinopathy and rubeotic glaucoma following cataract surgery. Eye 9: 728–732. 8849540 10.1038/eye.1995.185

[15] Kadonosono K, Matsumoto S, Uchio E, Sugita M, Akura J, et al (2001) Iris neovascularization after vitrectomy combined with phacoemulsification and intraocular lens implantation for proliferative diabetic retinopathy. Ophthalmic Surg Lasers 32 (1) 19–24. 11195738

[16] Douglas MJ, Scott IU, Flynn Jr HW (2003) Pars plana lensectomy, pars plana vitrectomy, and silicone oil tamponade as initial management of cataract and combined traction/rhegmatogenous retinal detachment involving the macula associated with severe proliferative diabetic retinopathy. Ophthalmic Surg Lasers Imaging 34 (4) 270–278. 12875454

[17] Honjo M, Ogura Y (1998) Surgical results of pars plana vitrectomy combined with phacoemulsification and intraocular lens implantation for complications of proliferative diabetic retinopathy. Ophthalmic Surg Lasers 29 (2) 99–105. 9507252

[18] Schiff WM, Barile GR, Hwang JC, Tseng JJ, Cekiç O, et al (2007) Diabetic vitrectomy influence of lens status upon anatomic and visual outcomes. Ophthalmology 114 (3) 544–550. 17169431 10.1016/j.ophtha.2006.08.017

[19] Adamis AP, Miller JW, Bernal MT, D'Amico DJ, Folkman J, et al (1994) Increased vascular endothelial growth factor levels in the vitreous of eyes with proliferative diabetic retinopathy. Am J Ophthalmol 118 (4) 445–450. 7943121 10.1016/s0002-9394(14)75794-0

[20] Funatsu H, Yamashita H, Noma H, Mimura T, Sakata K, et al (2004) Risk evaluation of outcome of vitreous surgery for proliferative diabetic retinopathy based on vitreous level of vascular endothelial growth factor and angiotensin II. Br J Ophthalmol 88 (8) 1064–1068. 15258026 10.1136/bjo.2003.032656 PMC1772284

[21] Tseng HY, Wu WC, Hsu SY (2007) Comparison of vitrectomy alone and combined vitrectomy, phacoemulsification and intraocular lens implantation for proliferative diabetic retinopathy. Kaohsiung J Med Sci 23 (7) 339–343. 17606428 10.1016/S1607-551X(09)70419-X PMC11918075

[22] Muselier A, Dugas B, Burelle X, Passemard M, Hubert I, et al (2010) Macular hole surgery and cataract extraction: combined vs. consecutive surgery. Am J Ophthalmol 150 (3) 387–391. 20615492 10.1016/j.ajo.2010.04.008

[23] Herrmann WA, Heimann H, Helbig H (2010) Cataract surgery: Effect on the posterior segment of the eye. Ophthalmologe 107 (10) 975–986. 20848110 10.1007/s00347-010-2236-2

